# Gradient adaptive sampling and multiple temporal scale 3D CNNs for tactile object recognition

**DOI:** 10.3389/fnbot.2023.1159168

**Published:** 2023-04-26

**Authors:** Xiaoliang Qian, Jia Meng, Wei Wang, Liying Jiang

**Affiliations:** College of Electrical and Information Engineering, Zhengzhou University of Light Industry, Zhengzhou, China

**Keywords:** tactile object recognition, gradient adaptive sampling, multiple temporal scale, 3D convolutional neural networks, MR3D-18 network

## Abstract

Tactile object recognition (TOR) is very important for the accurate perception of robots. Most of the TOR methods usually adopt uniform sampling strategy to randomly select tactile frames from a sequence of frames, which will lead to a dilemma problem, i.e., acquiring the tactile frames with high sampling rate will get lots of redundant data, while the low sampling rate will miss important information. In addition, the existing methods usually adopt single time scale to construct TOR model, which will induce that the generalization capability is not enough for processing the tactile data generated under different grasping speeds. To address the first problem, a novel gradient adaptive sampling (GAS) strategy is proposed, which can adaptively determine the sampling interval according to the importance of tactile data, therefore, the key information can be acquired as much as possible when the number of tactile frames is limited. To handle the second problem, a multiple temporal scale 3D convolutional neural networks (MTS-3DCNNs) model is proposed, which downsamples the input tactile frames with multiple temporal scales (MTSs) and extracts the MTS deep features, and the fused features have better generalization capability for recognizing the object grasped with different speed. Furthermore, the existing lightweight network ResNet3D-18 is modified to obtain a MR3D-18 network which can match the tactile data with smaller size and prevent the overfitting problem. The ablation studies show the effectiveness of GAS strategy, MTS-3DCNNs, and MR3D-18 networks. The comprehensive comparisons with advanced methods demonstrate that our method is SOTA on two benchmarks.

## 1. Introduction

The visual and tactile perception are two main ways that the robots perceive the world. The visual perception can only provide the appearance of an object to the robot, the physical characteristics of an object such as hardness, roughness, and texture, etc., must be obtained through the tactile perception. The tactile object recognition (TOR) is especially important for robots when the imaging condition is terrible or the object is deformable, and has important practical value in many robotic tasks, such as smart prosthetics (Wu et al., 2018), medical treatment (Liu et al., 2020), food industry (Philippe et al., 2004), refuse classification (Li et al., 2020), and post-disaster rescue (Gao et al., 2021), etc.

TOR can be roughly divided into two parts: acquiring tactile data and recognizing object category based on tactile data. First of all, the tactile sensors attached on the manipulator are used to acquire the tactile data (it is usually pressure data) of object. Afterwards, the CPU equipped in robots is employed to recognize object category according to tactile data, which is also the topic of this paper.

Recently, the deep learning technique has successfully applied in many fields (Qian et al., 2020, 2022; Ibrahim et al., 2022; Mao et al., 2022; Shi et al., 2022), and the deep learning based TOR methods can be classified into two classes according to whether the temporal information is involved: (1) TOR methods without temporal information, (2) spatiotemporal TOR methods.

Most of the traditional TOR methods belong to the first category. Gandarias et al. used an array of high-resolution pressure sensors to acquire tactile data, and then a convolutional neural network (CNN) is employed to obtain the features of tactile data, finally, the object categories are recognized by a trained support vector machine (SVM) (Gandarias et al., 2017). Sundaram et al. (2019) utilized a 32 × 32 pressure sensor array attached on a knitted glove to acquire tactile data, and then the multiple frames of tactile data were simultaneously imported into multiple CNNs for feature extraction and fusion, the final classification results were given by a softmax classifier. Other related works include: Liu et al. (2016) and Yi et al. (2022), etc.

Recently, the second category methods are the mainstream. A 5 × 5 pressure sensor array attached on a two-finger manipulator was used to acquire tactile frames (Zhang et al., 2018), each frame was then resized to a 1 × 25 vector and fed into the LSTM for feature extraction, afterwards, the extracted features at different sampling moments were assigned different weights via a self-attention module, finally, the weighted feature vectors were used for TOR. The stacks of tactile frames and tactile flow of which the computing scheme is similar to optical flow were used as dual input (Cao et al., 2018), and were extracted initial features by two residual orthogonal tiling convolutions (ROTConvs) branches, afterwards, the initial features were further refined by orthogonal tiling convolutions (OTConv), finally, the refined features were used to identify the object category through softmax classifier. A 28 × 50 pressure sensor array attached on a two-finger manipulator was used to acquire tactile data (Pastor et al., 2019), and then a 3D CNN was employed to acquire the time series features and accomplish the object recognition. Other related works include Cao et al. (2016), Funabashi et al. (2020), Bottcher et al. (2021), and Song et al. (2022), etc.

Some researchers have concluded that the tactile data sampled at different moment have a strong correlation and their changes contain important information about the shape, hardness, and roughness of object (Brayshaw et al., 2022; Li et al., 2022; Sun et al., 2022), consequently, the aforementioned second category methods are prominent, however, they still have two problems which are elaborated as follows.

The first problem of state-of-the-art (SOTA) methods lies in the sampling strategy. The SOTA methods usually adopt uniform sampling strategy to randomly select tactile frames from a sequence of frames, i.e., the time interval of neighbor frames is equal. The precondition of this strategy is that the importance of each tactile frame is equal, however, the opposite is true. As a matter of fact, the tactile frames are similar to each other and contain lots of redundant information in the untouching stage or stable grasping stage, by contrast, the tactile frames are more diversified and contain more useful information from the initial stage of touching object until the stable grasping. Consequently, adopting the uniform sampling strategy will face a dilemma, i.e., acquiring the tactile frames with high sampling rate will get lots of redundant data, while the low sampling rate will miss important information.

The second problem of SOTA methods lies in the generalization capability. The SOTA methods usually adopt single temporal scale to construct TOR model, which will induce that the generalization capability is not enough for processing the tactile data generated under different grasping speeds. As a matter of fact, the grasping speed of different robots is quite different, consequently, the time interval of neighbor tactile frame is variable. A smaller time scale is more suitable for the fast grasping, and vice versa. Therefore, using single temporal scale will lead to the poor generalization of TOR model.

To address the first problem, a gradient adaptive sampling (GAS) strategy is proposed and used to select the input tactile frames from all frames, which can adaptively determine the sampling interval in terms of the importance of tactile data to obtain the key information as much as possible under the condition of same number of input frames. To handle the second problem, a multiple temporal scale 3D CNNs (MTS-3DCNNs) model is proposed, which can extract multi-level deep features corresponding to different temporal scales for TOR, and the proposed model will have a better generalization capability for variable grasping speed in this way. Furthermore, the existing lightweight network ResNet3D-18 (Hara et al., 2018) is modified to prevent the overfitting problem and match the size of tactile data which is usually smaller than image data. The modified ResNet3D-18 network is denoted as MR3D-18 network and is used as the backbone network in the proposed MTS-3DCNNs model.

The main contributions are as follows:

1. A GAS strategy is proposed to address the information redundancy/loss problem induced by existing uniform sampling strategy.

2. A MTS-3DCNNs model is proposed to handle the problem that the generalization capability of existing TOR model is insufficient for processing the tactile data generated under different grasping speeds.

3. The lightweight network ResNet3D-18 is modified to obtain a MR3D-18 network which can match the tactile data with smaller size and prevent the overfitting problem.

## 2. Proposed methods

**Figure 1:**
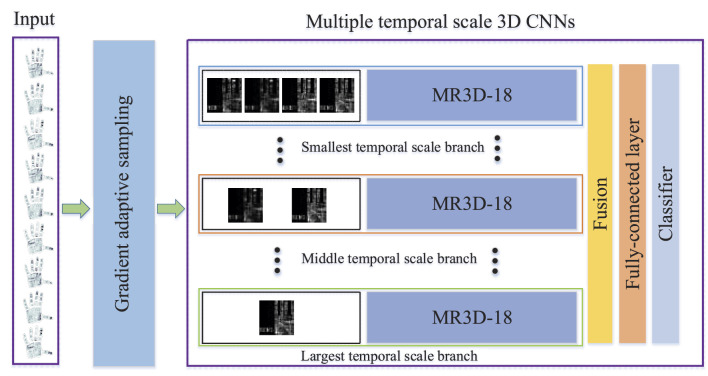
Framework of our method.

As shown in Figure 1, firstly, the input tactile frames are adaptively selected from all frames by using the GAS strategy, and are imported into the MTS-3DCNNs model. Secondly, the input tactile frames are downsampled with different temporal scales, and are fed into different network branches to extract the multiple temporal scale (MTS) deep features, where the MR3D-18 network is used as the backbone network. Finally, the fusing features of MTS deep features are employed to recognize the grasping object.

### 2.1. Gradient adaptive sampling strategy

**Figure 2:**
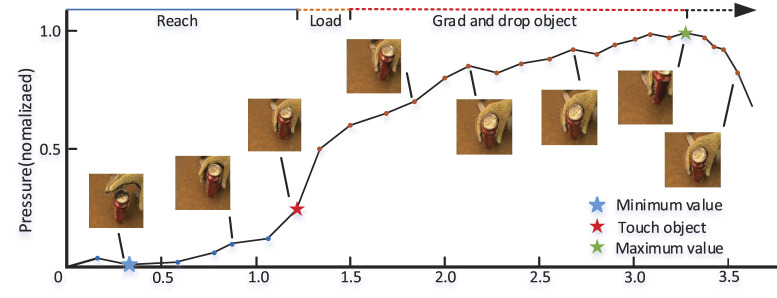
A typical interaction sequence of grasping objects by hand.

As shown in Figure 2, a typical procedure of grasping object can be roughly divided into three stages (Sundaram et al., 2019), which are denoted as “Reach”, “Load”, and “Grad and drop object”, respectively. In the first stage, the hand is gradually close to the object and the gesture is relatively fixed when the hand touch the object, consequently, the variation of pressure value is small and only the deformable pressure caused by the gesture can partially reflect the shape of the object in this stage. In the second stage, the contact area and pressure value between hand and object increase rapidly, consequently, the tactile frames are dramatically changed and contain lots of key characteristics, such as hardness, shape etc. In the third stage, the object is successively picked up and put down, and the average pressure value reaches its highest point and then decreases. The change speed of tactile frames in the third stage is between the first and second stages, and the tactile frames in third stage also contain some important characteristics. In summary, the order of three stages are stage 2, stage 3, and stage 1 in terms of importance, which is in accord with the order of the average gradient values of pressure values at each stage. Therefore, the proposed GAS strategy adaptively determines the sampling interval according to the gradient value of the tactile data. The details are as follows.

**Figure 3:**
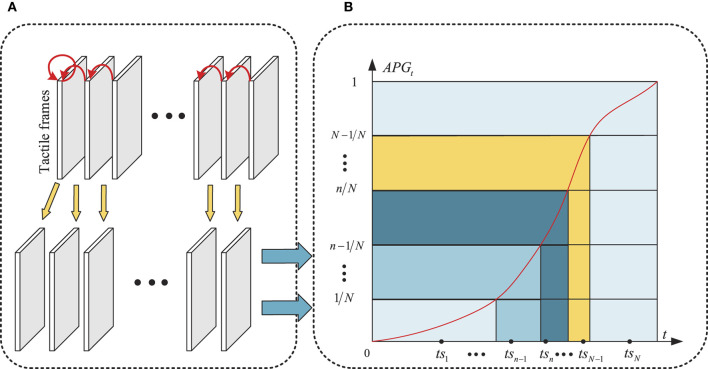
Illustration of GAS strategy. **(A)** Gradient representation of tactile frames, **(B)** Accumulative gradient distribution with respect to time.

As Illustrated in Figure 3A, the gradient representation of the tactile frames $FG=\left\{FG_{1},\ldots ,FG_{t},\ldots ,FG_{T}\right\}\in {\mathbb{R}}^{H\times W\times T}$ is formulated as:


(1) $$\{\begin{array}{l} FG_{t}=\left|F_{t}-F_{t-1}\right|,\;F_{t}\in D\in {\mathbb{R}}^{H\times W\times T},\;t\in \mathbb{Z},\;2\leq t\leq T\;\\ FG_{1}=0\; \end{array}$$

where *D* denotes the assemble of original tactile frames, *H, W*, and *T* separately indicate the height, width and number of the tactile frames, *F*_*t*_ denotes original tactile frame at moment *t*, and *FG*_*t*_ denotes the absolute value of gradient of two adjacent tactile frames at moment *t*.

The accumulative gradient distribution with respect to time is formulated as:


(2) $$\{\begin{array}{l} NFG_{t}=\frac{\sum_{x=1}^{H}\sum_{y=1}^{W}FG_{t}(x,y)}{\sum_{t=1}^{T}\sum_{x=1}^{H}\sum_{y=1}^{W}FG_{t}(x,y)},\;t\in \left[1,T\right]\\ AFG_{t}=\sum_{a=1}^{t}NFG_{a},\;AFG_{t}\in [0,1] \end{array}$$

where *NFG*_*t*_ denotes the normalized version of *FG*_*t*_, *FG*_*t*_(*x, y*) denotes the element in row *x* and column *y* of *FG*_*t*_, and *AFG*_*t*_∈[0, 1] denotes the accumulative gradient at moment *t*, i.e., *AFG*_1_ = 0, *AFG*_*T*_ = 1.

As shown in Figure 3B, the horizontal and vertical axes indicate *t* and *AFG*_*t*_, respectively, the red curve denotes the function curve of *AFG*_*t*_. The value range of *AFG*_*t*_, i.e., [0,1] is divided into *N* subintervals, i.e., {[0, 1/*N*], …, [*n*−1/*N*, *n/N*], ..., [*N*−1/*N*, 1]}, the *N* corresponding subintervals on the horizontal axis can also be obtained according to the function curve of *AFG*_*t*_, i.e., {[0, *si*_1_], …, [*si*_*n*−1_, *si*_*n*_], …, [*si*_*N*−1_, *T*]}, the final *N* sampling points are randomly selected from above *N* subintervals, i.e., *TS* = {*ts*_1_, *ts*_2_, …, *ts*_*N*_}, as shown in the black point in Figure 3B. Thus, the input tactile frames of following TOR model can be sampled from *D* according to *TS*, which are denoted as $F_{TS}=\left[F_{ts_{1}},\;F_{ts_{2}},\ldots ,\;F_{ts_{N}}\right]\in {\mathbb{R}}^{H\times W\times N}$.

As shown in Figure 3B, following the GAS strategy, the majority of *F*_*TS*_ focus on the subintervals where the gradient changes quickly.

### 2.2. Multiple temporal scale 3D CNNs for tactile object recognition

First of all, a MTS downsampling scheme is imposed on the *F*_*TS*_, and then the deep features of downsampled frames with different temporal scales are extracted through the MR3D-18 network, finally, the fused features of MTS deep features are used to recognize the grasping object.

#### 2.2.1. Multiple temporal scale downsampling

The MTS downsampling data *DF* = {*DF*_1_, ..., *DF*_*m*_, …, *DF*_*M*_} of input data *F*_*TS*_ can be obtained through following equation:


(3) $$\begin{array}{l} DF_{m}=DS(F_{TS},r_{m})\in {\mathbb{R}}^{H\times W\times N_{m}},\;m\in \mathbb{Z},\;1\leq m\leq M,\;\\ \;N_{m}=\left\lceil N\times r_{m}\right\rceil \end{array}$$

where *M* denotes the number of temporal scales and is quantitatively analyzed in Table 3, $r_{m}={\left(0.5\right)}^{m-1}$ denotes the downsampling ratio of *m*-th temporal scale, *DS*(·, ·) denotes the downsamling operation which is quantitatively analyzed in Table 3, *DF*_*m*_ denotes the downsampling data of *F*_*TS*_ at *m*-th temporal scale, *N*_*m*_ denotes the number of tactile frames contained in *DF*_*m*_.

#### 2.2.2. Feature extraction based on MR3D-18 network

As shown in Figure 1, the ${\left\{DF_{m}\right\}}_{m=1}^{M}$ are fed into the MR3D-18 network to obtain the MTS features {*SF*_1_, ..., *SF*_*m*_, …, *SF*_*M*_}. The MR3D-18 network is obtained by modifying the lightweight model ResNet3D-18. The motivation behind the modification includes two aspects: the size of tactile frame is usually smaller than the nature scene image which is the input of ResNet3D-18, and prevents the overfitting problem. Consequently, as shown in Table 1, the differences between MR3D-18 and ResNet3D-18 networks correspond to above two aspects and are given as follows.

**Table 1:** Comparison between ResNet3D-18 and MR3D-18 networks with a 32 × 32 input tactile frame.

**Layers**	**ResNet3D-18**		**MR3D-18**	
	**Filters**	**Output size**	**Filters**	**Output size**
Conv1	7 × 7 × 7, 64 stride 1, 2^2^	32 × 16^2^	7 × 7 × 7, 64 stride 1, 2^2^	32 × 16^2^
Pool	3 × 3 × 3 max stride 1, 2^2^	16 × 8^2^	—	32 × 16^2^
Res2	$\left[\begin{array}{c} 3\times 3\times 3,643\times 3\times 3,64 \end{array}\right]\times 2$ stride 1,1^2^	16 × 8^2^	$\left[\begin{array}{c} 3\times 3\times 3,643\times 3\times 3,64 \end{array}\right]\times 2$ stride 1,1^2^	32 × 16^2^
Dropout	—	16 × 8^2^	dropout rate=0.3	32 × 16^2^
Res3	$\left[\begin{array}{c} 3\times 3\times 3,1283\times 3\times 3,128 \end{array}\right]\times 2$ stride 2,2^2^	8 × 4^2^	$\left[\begin{array}{c} 3\times 3\times 3,1283\times 3\times 3,128 \end{array}\right]\times 2$ stride 2,2^2^	16 × 8^2^
Res4	$\left[\begin{array}{c} 3\times 3\times 3,2563\times 3\times 3,256 \end{array}\right]\times 2$ stride 2,2^2^	4 × 2^2^	$\left[\begin{array}{c} 3\times 3\times 3,2563\times 3\times 3,256 \end{array}\right]\times 2$ stride 2,2^2^	8 × 4^2^
Res5	$\left[\begin{array}{c} 3\times 3\times 3,5123\times 3\times 3,512 \end{array}\right]\times 2$ stride 2,2^2^	2 × 1^2^	$\left[\begin{array}{c} 3\times 3\times 3,5123\times 3\times 3,512 \end{array}\right]\times 2$ stride 2,2^2^	4 × 2^2^
Global average pooling				

1: MR3D-18 network removes a pooling layer from ResNet3D-18 network. As a matter of fact, the size of tactile frame usually does not exceed 32 × 32, consequently, the convolution operations in the top layers will require lots of zero filling if the size of features is reduced too much by pooling operation in the bottom layer. Therefore, removing a pooling layer can reduce the error caused by lots of zero filling.

2: MR3D-18 network adds a dropout layer to ResNet3D-18 network. The appearances of some objects with same category are quietly different with each other, and the grasping position of same object is different for each grasping moment, which leads to a high diversity of tactile data with same category. Consequently, the training of the 3D CNN is easily overfitting unless the manually labeled samples are enormous. Therefore, adding a dropout layer is necessary to enhance the generalization capability of MR3D-18 network. The layers in which the dropout layer is inserted and the deactivation rate are quantitatively analyzed in Table 2.

**Table 2:** Ablation study of MR3D-18 network in terms of top 1 score (%) on the MIT-STAG dataset.

**Dropout** **Model**	**0**	**0.1**	**0.2**	**0.3**	**0.4**	**0.5**
MR3D-18 (Res2)	80.30	80.70	81.27	**81.54**	79.25	76.73
MR3D-18 (Res3)	80.30	81.10	80.14	74.34	72.09	67.35
MR3D-18 (Res4)	80.30	81.29	78.34	78.97	78.23	75.28
ResNet3D-18 (Baseline)	74.49					

The bold font denotes the highest score.

#### 2.2.3. Object category recognition

The MTS features {*SF*_1_, ..., *SF*_*m*_, …, *SF*_*M*_} are firstly fused, and then the fused features successively pass through a FC layer and a softmax classifier to obtain the score vector, above procedure can be formulated as:


(4) $$\begin{array}{l} S=Softmax\left(FC\left(FUSION\left(SF_{1},\ldots ,SF_{m},\ldots ,SF_{M}\right)\right)\right) \end{array}$$

where *FUSION*(·) denotes feature fusion operation which is quantitatively analyzed in Table 3, *FC*(·) denotes the fully connected convolution operation, *Softmax*(·) denotes the softmax classification operation, *S*∈ℝ^*C*^ denotes the score vector of *F*_*TS*_, and *C* denotes the number of object categories. The category corresponding to the highest score in *S* is considered as the predicted object category.

**Table 3:** Ablation study of the proposed GAS and MTS-3DCNNs in terms of top 1 score (%) on the MTS-STAG dataset.

**Method**	**GAS**	**Downsampling**		***M***			***FUSION***(·)		**Top 1 score**
		**SubS**	**AvgP**	**1**	**2**	**3**	**Sum**	**Cat**	
A (baseline)				√					81.54
B		√			√			√	83.03
C		√			√		√		83.51
D		√				√		√	84.46
E		√				√	√		85.13
F	√			√					84.98
G	√	√			√			√	85.44
H	√	√			√		√		86.84
I	√	√				√		√	87.15
J	√	√				√	√		87.40
BP			√		√			√	83.60
CP			√		√		√		84.53
DP			√			√		√	85.64
EP			√			√	√		86.69
GP	√		√		√			√	87.05
HP	√		√		√		√		87.69
IP	√		√			√		√	87.95
JP (ours)	√		√			√	√		**88.81**

The *M* and *FUSION*(·) are defined in Equation (3) and (4), respectively. SubS and AvgP denote subsampling and average pooling operations, respectively. Sum and Cat denote summation and concatenation operations, respectively. The bold font denotes the highest score.

Aforementioned MTS-3DCNNs model is trained by the traditional cross entropy loss in an end-to-end manner.

In inference stage, the GAS is implemented when *T* = α*N*, α∈ℤ, i.e., GAS can be done even the sequence is not over. In other words, the object recognition result is updated when *T* = α*N*.

## 3. Experiment

### 3.1. Experiment setting

#### 3.1.1. Datasets

The MIT-STAG (Sundaram et al., 2019) and iCub (Soh et al., 2012) datasets are employed to verify the effectiveness of our method. The MIT-STAG dataset includes 88269 valid tactile frames with 27 categories, and the size of each frame is 32 × 32. The tactile data are acquired by a 32 × 32 pressure sensor array attached on a knitted glove, and the 27 categories include 26 common objects and empty hand. To balance the number of frames of each category, 36531 frames (1353 frames per category) are selected as the training set, and 16,119 frames (597 frames per category) are selected as the testing set. The MIT-STAG is a large scale dataset, and lots of tactile frames belonging to different category have similar appearance characteristics, obviously, it brings great challenge for TOR methods.

The iCub dataset includes 2,200 tactile frames with 10 categories, and each category includes 220 tactile frames of which the size is 5 × 12. The tactile frames are acquired through an iCub humanoid robot platform. The platform has two anthropomorphic dexterous hands with 5 fingers (20 joints, 9 freedom degrees), and each fingertip is equipped with 12 capacitive pressure sensors. The training set and testing set include 1,320 frames (132 frames per category) and 880 frames (88 frames per category), respectively.

#### 3.1.2. Evaluation metrics

The top 1 score, kappa coefficient (KC) and confusion matrix are utilized to evaluate the effectiveness of our method, which are commonly used metrics for the TOR task.

#### 3.1.3. Implementation details

All of the input data are resized to 32 × 32. The ResNet3D-18 pre-trained on Kinetics400 (Carreira and Zisserman, 2017) is adopted as the backbone network of MTS-3DCNNs. The stochastic gradient descent (SGD) is adopted as the optimization algorithm, where the weight decay and momentum are set to 0.0001 and 0.9, respectively. The batchsize is set to 32 and 8 for the MIT-STAG and iCub datasets, respectively. The number of epochs is set to 50. The initial learning rate is set to 0.001, and it becomes 10% of last stage after every 10 epochs. The experiments are implemented in the PyTorch framework, and runs on single NVIDIA GeForce RTX 2080Ti@11GB GPU.

### 3.2. Ablation study

#### 3.2.1. Ablation study of MR3D-18 network

To verify the effectiveness of MR3D-18 network, the MR3D-18 network is compared with original ResNet3D-18 network (baseline method), furthermore, the MR3D-18 networks with different dropout configuration are also compared with each other. The MR3D-18 (Res2), MR3D-18 (Res3), and MR3D-18 (Res4) denote the dropout layer is added after Res2, Res3 and Res4 layers, respectively. The dropout (0~0.5) denotes the deactivation rate of dropout layer. It is worth noting that the MR3D-18 (dropout = 0) denotes that only a pooling layer is removed from the ResNet3D-18 network.

As shown in Table 2, the comparison between MR3D-18 (dropout = 0) and ResNet3D-18 shows that the top 1 score is increased by 5.81% via removing a pooling layer. The comparisons between MR3D-18 networks with different dropout configuration show that the MR3D-18 network achieves the best performance when the dropout layer is added after Res2 and the deactivation rate is set to 0.3, and the top 1 score is increased by 7.05% compared with ResNet3D-18 network.

In a word, the MR3D-18 network is superior to the ResNet3D-18 network for the TOR, i.e., the proposed modification scheme of ResNet3D-18 network is effectiveness. The MR3D-18 (Res2) with deactivation rate equals 0.3 is adopted in the following experiments.

#### 3.2.2. Ablation study of GAS and MTS-3DCNNs

To verify the effectiveness of GAS strategy and MTS-3DCNNs, 18 methods with different configurations are compared with each other, which are denoted as A~JP, respectively. The method F~J and GP~JP adopt the GAS, the rest is not. The downsamping strategy adopted by method BP~JP is average pooling operation, the rest methods adopt subsampling operation except method A and F. The method A and F do not use the MTS scheme, i.e., *M* = 1. The method B, BP, C, CP, G, GP, H, and HP use two temporal scales (*M* = 2), and the method D, DP, E, EP, I, IP, J, and JP use three temporal scales (*M* = 3). The feature fusion strategy, i.e., *FUSION*(·), adopted by method C, CP, E, EP, H, HP, J, and JP is summation operation, the rest methods adopt the concatenation operation except method A and F. The method A does not adopt the GAS and MTS scheme, therefore, it is denoted as the baseline method.

As shown in Table 3, the comparisons between A~E and F~J (or BP~EP and GP~JP) demonstrate that using the GAS can apparently improve the performance of TOR. The comparisons between A and B~E, BP~EP (or F and G~J, GP~JP) show that the MTS scheme is superior to the single temporal scale scheme. The comparisons between B, C and D, E (or G, H and I, J, or BP, CP and DP, EP, or GP, HP and IP, JP) show that the performance is better when *M* = 3. The comparisons between B and C (or D and E, or G and H, or I and J, etc.) show that the summation operation is a more appropriate feature fusion strategy. The comparison between B and BP (or C and CP, or D and DP, or E and EP, etc.) shows that average pooling is a more appropriate downsampling operation. The comparison between A and JP demonstrates that the top 1 score of baseline method can be increased by 7.27% via using both GAS and MTS scheme.

In a word, the proposed GAS and MTS scheme are effective.

### 3.3. Comparisons with state-of-the-art methods

To further evaluate the effectiveness of our method, it is compared with some state-of-the-art methods in terms of top 1 score, KC and confusion matrix. As shown in Tables 4, 5, Sundaram et al. (2019) provided the source codes, the rest comparison methods only provided the experimental results on one dataset. The comparison results on the MIT-STAG and iCub datasets are shown in Tables 4, 5, respectively.

**Table 4:** Comparisons with the state-of-the-art methods in terms of top 1 score (%) and KC (%) on the MIT-STAG dataset.

**Method**	**Top 1 score**	**KC**
Sundaram et al., 2019	72.38	71.35
Wang et al., 2021	72.00	70.96
Zhang et al., 2021	80.09	79.31
Sharma, 2022	81.82	81.13
Ours	**88.81**	**88.53**

The bold font denotes the highest score.

**Table 5:** Comparisons with the state-of-the-art methods in terms of top 1 score (%) and KC (%) on the iCub dataset.

**Method**	**Top 1 score**	**KC**
DS (Soh and Demiris, 2014)	98.5	98.4
GS (Soh and Demiris, 2014)	98.9	98.8
Soh et al., 2012	99.3	99.2
Sundaram et al., 2019	99.5	99.4
Ours	**100**	**1**

The bold font denotes the highest score.

**Figure 4:**
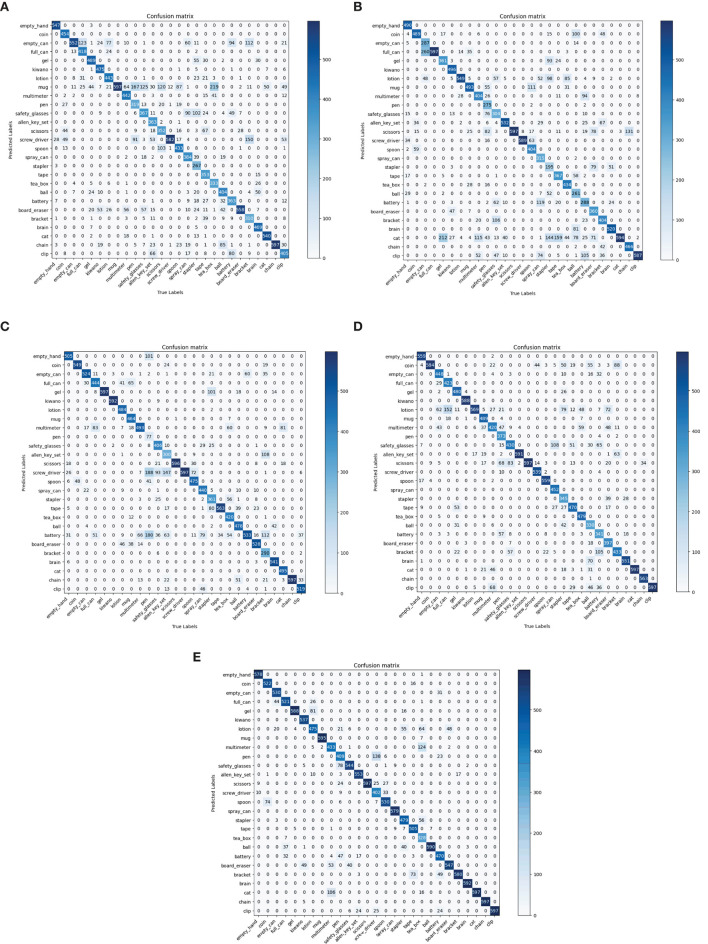
Comparisons with state-of-the-art models in terms of confusion matrix on the MIT-STAG dataset. **(A)** Sundaram et al., 2019, **(B)** Wang et al., 2021, **(C)** Zhang et al., 2021, **(D)** Sharma, 2022, **(E)** Ours.

As shown in Table 4, the top 1 score of the proposed method surpasses the Sundaram et al. (2019), Wang et al. (2021), Zhang et al. (2021), and Sharma (2022) by 16.43, 16.81, 8.72, and 6.99%, which indicates that our method has the highest classification accuracy on the MIT-STAG dataset. Meanwhile, the proposed method of KC surpasses the Sundaram et al. (2019), Wang et al. (2021), Zhang et al. (2021), and Sharma (2022) by 17.18, 17.57, 9.22, and 7.4%, which indicates that our method has the lowest degree of confusion which can also be seen in Figure 4.

**Figure 5:**
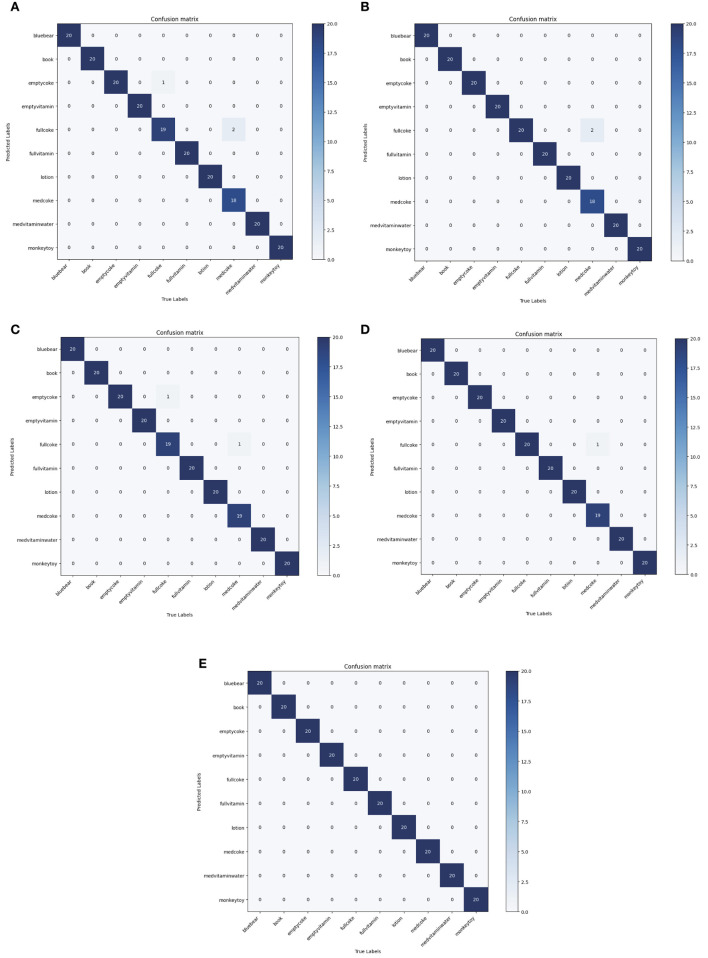
Comparisons with state-of-the-art models in terms of confusion matrix on the iCub dataset. **(A)** DS (Soh and Demiris, 2014), **(B)** GS (Soh and Demiris, 2014), **(C)** Soh et al., 2012, **(D)** Sundaram et al., 2019, **(E)** Ours.

As shown in Table 5, the top 1 score of the proposed method surpasses the DS (Soh and Demiris, 2014), GS (Soh and Demiris, 2014), Soh et al. (2012), and Sundaram et al. (2019) by 1.5, 1.1, 0.7, and 0.5%, which indicates that our method has the highest classification accuracy on the iCub dataset. Meanwhile, the proposed method of KC surpasses the DS (Soh and Demiris, 2014), GS (Soh and Demiris, 2014), Soh et al. (2012) and Sundaram et al. (2019) by 1.6, 1.2, 0.8, and 0.6%, which indicates that our method has the lowest degree of confusion which can also be seen in Figure 5.

In summary, our method has the best performance on the two datasets, and the relative superiority is more obvious on the more challenging MIT-STAG dataset, which further validate the effectiveness of our method.

## 4. Conclusion

A novel GAS strategy was proposed to address the problem that the uniform sampling strategy lead to the information redundancy or loss, which could adaptively determine the sampling interval according to the gradient value of the tactile frames, consequently, the key information of tactile frames could be acquired as much as possible under the condition of same number of input frames. A novel MTS-3DCNNs model was proposed to handle the problem that the generalization capability of existing TOR model was insufficient for processing the tactile frames generated under different grasping speeds. The MTS-3DCNNs model downsampled the input tactile frames with different time scale and extracted the MTS deep features, and the fused features were employed to recognize the grasping object. Consequently, the generalization capability of TOR model could be improved in this way for variable grasping speed. Furthermore, the lightweight network ResNet3D-18 is modified to obtain a MR3D-18 network which can match the tactile data with smaller size and prevent the overfitting problem. The ablation studies of MR3D-18 network, GAS strategy and MTS-3DCNNs model demonstrated that MR3D-18 network could give better performance than original ResNet3D-18 network and using the GAS strategy and MTS-3DCNNs model could effectively improve the performance of TOR model. The comparisons with advanced methods on the MIT-STAG and iCub datasets show that the overall performance of our method was SOTA.

## Data availability statement

The original contributions presented in the study are included in the article/supplementary material, further inquiries can be directed to the corresponding authors.

## Author contributions

XQ contributed to the key innovation and revised this paper. JM designed and debugged the codes of proposed method and wrote the draft. WW as the team leader, was responsible for the arrangement of overall work. LJ contributed to the revision of this paper. All authors contributed to the article and approved the submitted version.
